# Adverse Outcome Pathways can drive non-animal approaches for safety assessment

**DOI:** 10.1002/jat.3165

**Published:** 2015-05-05

**Authors:** Natalie Burden, Fiona Sewell, Melvin E Andersen, Alan Boobis, J Kevin Chipman, Mark T D Cronin, Thomas H Hutchinson, Ian Kimber, Maurice Whelan

**Affiliations:** a NC3RsGibbs Building, 215 Euston Road, London, NW1 2BE, UK; b Hamner Institutes for Health SciencesResearch Triangle Park, North Carolina, 27709, USA; c Imperial College London, Hammersmith CampusDu Cane Road, London, W12 0NN, UK; d University of Birmingham, School of BiosciencesEdgbaston, Birmingham, B15 2TT, UK; e Liverpool John Moores University, School of Pharmacy and Biomolecular SciencesByrom Street, Liverpool, L3 3AF, UK; f Plymouth University, School of Life SciencesDrake Circus, Plymouth, PL4 8AA, UK; g University of ManchesterOxford Road, Manchester, M13 9PT, UK; h European Commission Joint Research Centre, EU Reference Laboratory for Alternatives to Animal Testing (EURL ECVAM)20127, Ispra (VA), Italy

**Keywords:** chemical, safety assessment, risk, pathway, AOP, mechanism of action, mode of action, toxicity, 3Rs

## Abstract

Adverse Outcome Pathways (AOPs) provide an opportunity to develop new and more accurate safety assessment processes for drugs and other chemicals, and may ultimately play an important role in regulatory decision making. Not only can the development and application of AOPs pave the way for the development of improved evidence-based approaches for hazard and risk assessment, there is also the promise of a significant impact on animal welfare, with a reduced reliance on animal-based methods. The establishment of a useable and coherent knowledge framework under which AOPs will be developed and applied has been a first critical step towards realizing this opportunity. This article explores how the development of AOPs under this framework, and their application in practice, could benefit the science and practice of safety assessment, while in parallel stimulating a move away from traditional methods towards an increased acceptance of non-animal approaches. We discuss here the key areas where current, and future initiatives should be focused to enable the translation of AOPs into routine chemical safety assessment, and lasting 3Rs benefits. © 2015 The Authors. *Journal of Applied Toxicology* published by John Wiley & Sons Ltd.

This article explores how the development and application of Adverse Outcome Pathways (AOPs) could benefit the science and practice of chemical safety assessment, with a particular focus on how their use in practice could reduce reliance on traditional animal toxicity tests. This includes discussion of the key areas where current and future initiatives should be focused to enable the translation of AOPs into routine chemical safety assessment, and lasting 3Rs benefits.

## Introduction

**Figure 1:**
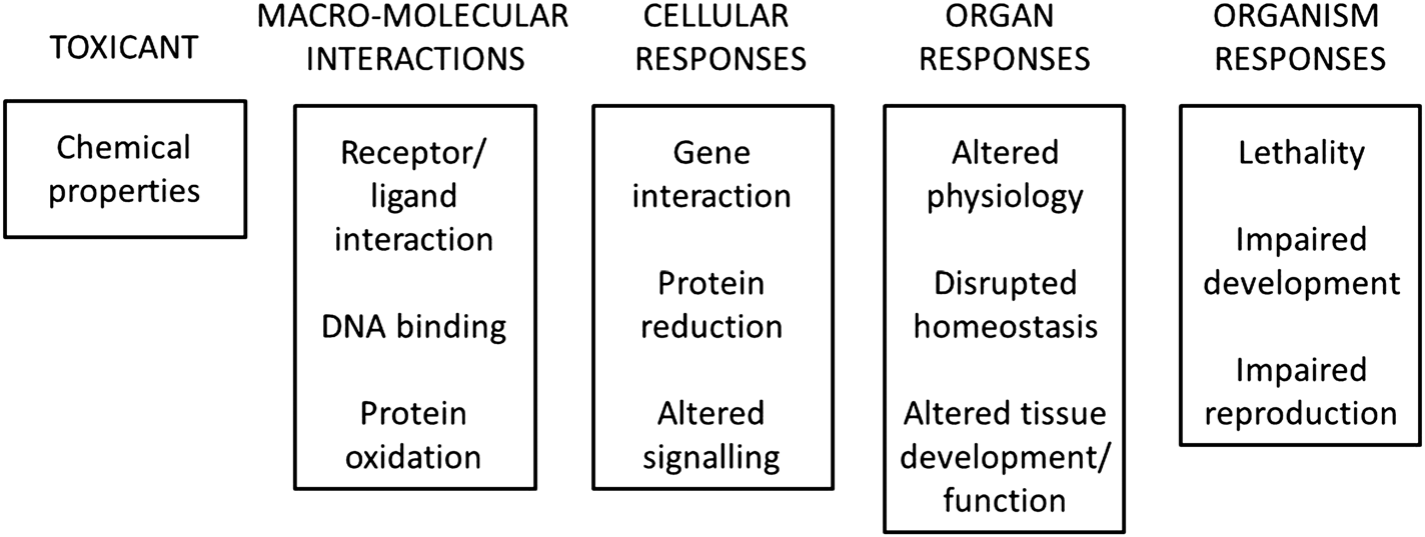
Schematic representation of the Adverse Outcome Pathways (AOP) with reference to examples of different pathways (adapted from an OECD figure, found at http://www.oecd.org/chemicalsafety/testing/adverse-outcome-pathways-molecular-screening-and-toxicogenomics.htm).

The use of a mechanistic ‘Adverse Outcome Pathway’ (AOP) approach has been highlighted in recent years as having the potential to improve chemical and drug safety assessment. AOPs support an understanding of how perturbation of normal biology can lead to an adverse outcome, by linking a molecular initiating event (MIE) for a drug or other chemical to an apical endpoint and subsequent organism/population effects, through a transparent, scientifically proven causal chain of events (Fig.1).

The AOP concept is not new and has been described to varying extents under a number of guises such as ‘toxicity pathways’, ‘mode of action’ and ‘mechanism of action’ (NRC, 2007; Wilkinson *et al*., 2000; Sonich-Mullin *et al*., 2001). There is considerable overlap in these terms and to some degree their definitions converge, with all terms focused on understanding the underlying biological mechanisms and pathways associated with the development of adverse effects. The critical defining factor of an AOP is that it anchors the MIE to an adverse outcome, which is of importance within a regulatory context (i.e. the identification of adverse effects at an organism or population level). Therefore, an AOP must contain an appropriate level of information regarding the causal key events that link the MIE and an adverse effect. It is these unique properties which mean that AOPs could be of tangible benefit to the development of new strategies for the safety assessment of both drugs and other chemicals. Despite the fact that pharmaceuticals differ from chemicals encountered in the workplace and environment in terms of their usage and exposure, and the need to offset risk against benefit, it is apparent that AOPs offer important benefits in both areas.

AOPs are being developed to different extents worldwide. However, there is a continuing debate surrounding what is understood by the term AOP, and their intended use and application. It has been recognized that to realize their widespread use in practice, the adoption of an explicit, consensus-led framework under which AOPs are developed and applied is crucial. In addressing this, the Organisation for Economic Co-operation and Development (OECD) AOP Development Programme (www.oecd.org/chemicalsafety/testing/adverse-outcome-pathways-molecular-screening-and-toxicogenomics.htm) has led to the establishment of such a framework. AOPs not only have the potential to transform safety assessment, but in the longer term offer benefits associated with a reduced reliance on *in vivo* testing in this area. Furthermore, moving away from the traditional animal models could drive an increase in the predictivity of toxicity testing. Important in this respect is an increased focus on consideration of species-relevant biology, for example through the use of human cell-based systems, and through the potential address more effectively aspects of inter-individual variability.

This article explores the opportunities that the development and implementation of the AOP framework offers the toxicology community, with a focus on the potential to reduce and replace the use of animals in regulatory human and environmental safety assessment, while recognizing the hurdles that must be overcome in the coming years before these opportunities can be exploited to their maximum potential.

### How Will the Application of AOPs Improve the Science and Practice of Safety Assessment, while Reducing our Reliance on Animal Tests?

AOPs are essentially a systematic way to organize, integrate and communicate mechanistic knowledge and information; their application holds great promise to increase the utilization of non-animal test methods that typically provide mechanistic information, to the benefit of the process of safety assessment, providing that information is used and interpreted prudently. Once developed, AOPs can be applied in two different ways: (i) to explain an observed adverse outcome in terms of an MIE and key events (a ‘top-down’ approach, which is reliant on *in vivo* toxicology); or (ii) to predict the likely adverse outcome after determination of an MIE and downstream key events (or ‘bottom-up’ approach, which employs non-animal methods). A targeted focus on applying the latter would enable a shift in the practice of safety assessment away from the traditional manner of measuring apical endpoints, towards the use of biological information generated using *in silico* and *in vitro* methods to predict whether a chemical causes specific perturbations, which we are confident would lead to apical responses. As AOPs describe the sequence of key events that lead to adversity at different levels of biological organisation, high throughput *in vitro* approaches (such as reporter gene assays that reflect specific molecular and cellular events) can be utilized to indicate toxic potential. In this way, AOPs could provide a bridge between non-animal methods and systems toxicology, which could well improve the discipline of non-animal based safety assessment. There is also the pragmatic recognition within the toxicology community that alternative methods will not replace *in vivo* methods on a like-for-like basis, and therefore AOPs will be used to form part of the scientific basis that informs and directs integrated approaches to testing and assessment (IATA; Patlewicz *et al*., 2014).

AOPs also provide the opportunity to extrapolate effects between species, qualitatively and quantitatively, through the identification of conserved MIEs, key events and key event relationships. The science of safety assessment would, therefore, be enhanced if AOPs could be used to extrapolate experimental outcomes accurately in populations of organisms in the environment to responses observed in humans. Furthermore, the categorical demonstration of conservation across species may decrease the numbers of different species which must be used in toxicity testing, including higher-order species such as non-human primates, and would have obvious benefits in improving the accuracy of ecotoxicological assessments. It is important to emphasize that accurate and relevant data will be required for progress towards the development and application of truly relevant methods. This might include building databases to share information on cross-species conservation.

The ongoing, iterative nature of the AOP development process means that gaps in our knowledge and the need for further research will be highlighted. Identification of the key data gaps could help to drive forward advances in non-animal approaches to help fill these gaps. There is also the opportunity for AOPs to drive novel approaches to hazard characterisation, e.g. through the development of mechanism-based biomarkers. The utility of AOPs is apparent not only in a regulatory setting – they also have potential to be used during early screens, to identify compounds/ substances that are likely to be associated with an unwanted adverse effect. There is certainly scope to use the AOPs that are in early development (i.e. less comprehensively described), in this context. Such approaches could be used for compound selection or product prioritisation, and would potentially reduce the amount of later *in vivo* testing carried out and reduce the severity of any responses during such testing. However, for their ultimate application in safety assessment, the methods used must be capable of providing quantifiable readouts which are informative of the ‘tipping points’ that propagate the pathways, and we must be confident that the tools and measures used are appropriate and fit-for-purpose.

### What is Needed to Realise the 3Rs Benefits of the AOP Approach?

Although laudable in its intentions, the development of AOPs themselves is not enough to achieve a shift towards more mechanistic, animal-free predictions of toxicity. To have genuine utility and impact on the replacement, refinement and reduction of animals – the 3Rs – they must be organized and applied under the auspices of the framework, which has been designed to enable coherency and to be accessible across the relevant scientific disciplines. It provides a systematic and structured way of capturing, curating and evaluating mechanistic knowledge, to render it practicably useable by a variety of non-toxicology-expert users. Tools that shape this framework are now available, including the AOP Wiki and Effectopedia, which can be found within the AOP Knowledge Base (a hub established by the OECD in collaboration with the US Environmental Protection Agency (EPA), the European Commission Joint Research Centre (JRC) and the US Army Engineer Research and Development Center (ERDC); https://aopkb.org/). These platforms represent an evolution in the development of AOPs that enable the organized collection and sharing of knowledge through international crowd-sourcing, to facilitate the transparent building of content within the AOPs currently included in the OECD’s AOP Workplan. Final acceptance of the AOPs after this development process will require a more formal procedure, including independent critical review. In this way, AOPs will be developed and used in a transparent manner. Transparency in AOP development is a critical issue, particularly when considering their potential application for regulatory purposes. Such transparency will also facilitate interoperability across different areas of human toxicology and ecotoxicology, thereby facilitating widespread, global applicability. The AOP framework further provides the opportunity to link different research programmes, particularly those which focus on the avoidance of *in vivo* testing. Examples of these include the EU SEURAT-1 initiative (a research programme funded jointly by the European Commission and the cosmetics industry, represented by Cosmetics Europe) to fill current gaps in scientific knowledge and accelerate the development of non-animal test methods in the complex area of repeated dose toxicity (www.seurat-1.eu); and the US Tox21 programme (www.epa.gov/ncct/Tox21), which pools resources from governmental organisations (NIH-NCATS, NIH-NIEHS, EPA and FDA) to use robotics technology to screen thousands of chemicals for potential toxicity, to use screening data to predict the potential toxicity of chemicals, and to develop a cost-effective approach for prioritizing the thousands of chemicals that have not undergone toxicity testing.

There are four focus areas that will be key to the successful exploitation of the AOP framework in order for it to genuinely impact on toxicology and safety assessment practice and, in turn, the 3Rs.

#### Exploitation of available information and data

There is generally no need to generate new animal data to begin building AOPs, as much of the mechanistic knowledge required is already available within the peer-reviewed literature. AOPs offer an effective means to distil out the essential features of this mechanistic information and make the knowledge more accessible. However, a paradigm shift may be needed to encourage scientists to share unpublished data more openly. There is also a need to increase awareness within the community about what data are already publicly available, e.g. the dissemination of information regarding accessible databases.

#### Harmonization of approaches

The inherent variation in the expanse and resolution of different AOPs presents a challenge, particularly with regards to the differing levels of description, depending on the available knowledge and the nature of the pathway itself. Therefore, in order for them to be successfully applied in routine practice, a consensus agreement is necessary on what level of information is required before an AOP is deemed useable, or alternatively the widespread acceptance that the level of detail needed will vary on a case-by-case basis and agreement reached on what the minimum information requirements would be for each scenario.

Above all, it is important that the data supplied are reliable and that there is sufficient supporting evidence to prove linkage at each step of the pathway. These are not new questions – for example, see Meek *et al*. (2014) – and a consensus is emerging (OECD, 2013, 2014). Although only a certain minimal level of information may be required to constitute an AOP, this should not discourage the development of more comprehensive AOPs. Furthermore, AOPs should be viewed as dynamic and evolving – an AOP is never perfect or absolutely finalized – and it can be constantly updated to ‘fill in the gaps’ and improve understanding. It is important that all efforts in this area align with a harmonized and consistent approach.

The development of an internationally agreed approach for the development of AOPs, under the OECD framework, provides a clear methodology for informing the Test Guidelines Programme towards the identification and prioritisation of new *in vitro* test methods that are candidates to become OECD Test Guidelines; the OECD QSAR Toolbox Project for the identification of new methods/profilers for grouping chemicals; and OECD Hazard Assessment activities for the development of IATA, for defined hazard endpoints. It is also more likely that regulatory acceptance of non-animal (vertebrate) methods will be achieved if there has been an agreement early-on around appropriate levels of information required to build and apply an AOP, and if regulators are involved in this process.

#### Fostering of collaborations

The focus on establishing the underlying biological mechanisms and key events in order to build AOPs requires input across multiple scientific disciplines, providing a communication tool and an opportunity to bring together technologies and understanding across the wider scientific community, thus reaching out to new fields and creating collaboration opportunities that were not available before. This could help to catalyse the design of informative non-animal methods for toxicity testing, by identifying novel applications for technologies such as *in silico*, QSAR, high throughput screening and ‘omics approaches, as well as to provide a platform for discussions among basic scientists, regulators, risk assessors and risk managers and industry. In addition, it is possible that the information gained from AOPs could be utilized by mathematical and computational modellers to produce more quantitative AOPs, where the key event forms the basis of quantitative dose-response models, and could provide more predictive information than the dose-responses for apical endpoints. The development and success of the framework will depend on buy-in and acceptance from multiple-disciplines, including input from regulators when deciding how this could be used for safety assessment within a regulatory context.

#### Engaging regulators in the acceptance of mechanistic data and alternative methods

Regulatory acceptance is a major roadblock, not only for AOPs, but in many cases for non-animal toxicity data if we are to use these approaches in the wider setting and if we expect to see real 3Rs benefits. There has been some inconsistency in the use of mechanistic/AOP information within safety assessment amongst regulators, e.g. the cancer risk assessment of TCDD (Popp *et al*., 2006); the time is now right to engage regulators in these discussions, and to identify where and how the differences of opinion come about. There are several questions relevant to the use of AOPs in a regulatory context that must be to be answered, including: What are the weight-of-evidence considerations necessary before an AOP can be accepted? Would reduced inter-species extrapolation factors on the basis of an AOP be accepted? Moreover, would an AOP based solely on *in vitro* or computational data ever be acceptable as a basis for chemical safety assessment?

### Towards the Use of AOPs in Practice

Now that the AOP framework is here, and there is subsequently much investment in AOP development, we need to consider how best to ensure that they will really be utilized and applied in practice and that the efforts thus far do not prove futile.

First, the development of AOPs should take into account their intended application. For use in a regulatory context, it is necessary to maintain a focus on the regulatory goal of the activity and to foster an appreciation that endpoints other than apical endpoints could be used for safety assessment. This will require discussion and agreement amongst relevant organisations worldwide. It is also important that the bigger picture is not ignored. For example, it is necessary that the conditions that propagate the AOP (i.e. causality) are defined, and that toxicokinetics and exposure/dose relationships are considered, as these could alter the extent to which the normal biological process is perturbed, the consequent course of events and the final adverse outcome. Efforts must be made around understanding the quantitative linkages between downstream key events, as well as accounting for inter-individual variability, and variations in key event-dose relationships.

As previously mentioned, efforts are already underway to incorporate AOPs into the process of informing and/or directing IATAs. Other ways in which AOPs could be applied for safety assessment should also be considered; for example, there is potential to use them to justify or contribute towards decisions that are made in a regulatory setting, such as in the choice of appropriate uncertainty factors or chemical-specific adjustment factors (CSAFs). Further possible applications include their use in the identification of appropriate dose metrics within safety assessment, as well as in the grouping of chemicals for cumulative safety assessment, and in category formation and read-across.

Finally, as previously alluded to, it is possible that less well-defined AOPs would be adequate for de-prioritizing molecules during the development process, through their use in screening level assessments and prioritization decisions, for example the use of potency for the MIE to rank chemicals. In this way, AOPs could be used to improve the early-screens employed for go/no-go decisions, and thus decrease the numbers of failures of compounds at later stages by helping to prioritise chemicals taken forward for more detailed assessment. There could also be the option to identify potential adversity from complex mixtures where chemical analysis alone is insufficient. One impact of their use at early development stages could be to cut down on numbers of molecules going through to *in vivo* test stages, or in ecotoxicology to help focus on invertebrate models, and could thus have further benefits such as the saving of significant time and resource. If used in this way, the data generated could be compared with those from the traditional regulatory toxicity tests, to help towards validation efforts, to build regulatory confidence, and to inform applicability domains.

Efforts are now underway to enable the practical application of the most well-defined AOP, that of skin sensitization initiated by covalent binding of chemical to proteins (OECD, 2012). This is a pertinent example of how the assimilation of knowledge about underlying chemical and biological processes can be used to enable the distillation of the mechanisms into a number of key events that are proven to result in an adverse health effect in humans. The establishment of this AOP will allow for the exploitation of the various *in vitro* methods that have been developed to assess skin sensitization, and illustrates how collaborative efforts by the scientific community through a coordinated framework could lead to the incorporation of a mechanistic understanding into an effective risk assessment process. The next stage currently underway is the utilization of this AOP to drive development of an IATA; the aim now will be to ensure that current framework can streamline efforts to progress AOPs in other areas, to the point where they can make a meaningful contribution to safety assessment.

## Conclusion

It is clear that the well-considered use of AOPs has the potential to drive positive change in toxicology towards a reduced reliance on predictions made using vertebrate animal models and on the measurement of apical toxicity endpoints *per se*. The concept itself, and the widespread international uptake, is already stimulating the development of novel tools and screening methods and driving real innovation within human toxicology and ecotoxicology. However, for the true gains and a genuine 3Rs impact to be realised, we must continue to make concerted efforts to collect, integrate and organise data from relevant sources, across scientific disciplines, to realise the benefits of having a coherent framework. This will also require regulatory input, to provide advice on how information from AOPs can be used in decision making.

Even if AOPs in themselves do not come to fruition for use in a regulatory setting, the current large-scale investment into their development will undoubtedly accelerate the progress, understanding and use of non-animal methods, and could go some way towards convincing regulators to move away from relying for the most part on the traditionally used animal tests. There is no doubt that AOPs have a place in the prediction of chemical and drug toxicity; it is now time for the safety assessment community to decide how and where their application can have the greatest scientific and 3Rs benefits, and work together to enable this transition.

## Conflict of Interest

The Authors did not report any conflict of interest.
